# Best practices for physiological data collection in youth with autism and co-occurring mental health diagnoses: Implications for human-animal intervention research

**DOI:** 10.1016/j.mex.2025.103284

**Published:** 2025-03-27

**Authors:** Cory M. Smith, Katharine Weimann, Madison Widick, Tamara Merritt, Hannah Christensen, Matthew Siegel, Zhaoxing Pan, Robin L. Gabriels

**Affiliations:** a Baylor University Waco, TX Robbins College of Health and Human Sciences, United States; b University of Colorado Anschutz Medical Campus and the Children's Hospital Colorado Aurora, CO, United States of America; c Hearts & Horses, Inc. Loveland, CO, United States of America; d University of Montana Missoula, MT, United States of America; e Boston Children's Hospital, Department of Psychiatry & Behavioral Sciences, United States of America; f Tufts University School of Medicine, Departments of Psychiatry & Pediatrics, United States of America

**Keywords:** Wearable Physiological Monitoring during Equine-Assisted Services., Electrocardiogram, Heart rate variability, Galvanic skin response, Electrodermal activity, THR, Horseback riding, Stress response, Team dynamics, ASD, Human animal interventions

## Abstract

The purpose of this paper is to serve as a catalyst for the human-animal interaction research field to improve scientific rigor and accelerate the knowledge of field-based physiological responses during equine-assisted services in youth with autism spectrum disorder. This paper outlines the best practices for collecting and analyzing electrocardiogram and electrodermal activity in youth with autism spectrum disorder, utilized during a 10-week therapeutic horseback riding intervention.

• Motivation strategies such as device choice, reward systems, and a visual schedule should be implemented to improve participant compliance. In addition, devices should be secured to the participant following implementation of appropriate desensitization techniques.
• Time-domain heart rate variability analyses are more appropriate during therapeutic horseback riding data collection compared to frequency-domain approaches. For electrodermal activity, tonic responses should be assessed as opposed to phasic analyses.
• An effective data monitoring team including the Data Collection Research Personnel, Site Principal Investigator, Physiologist, and Therapeutic Riding Center Intervention Lead are key to increasing the quality of usable data in equine-assisted service research environments.

## Graphical abstract

**Figure:**
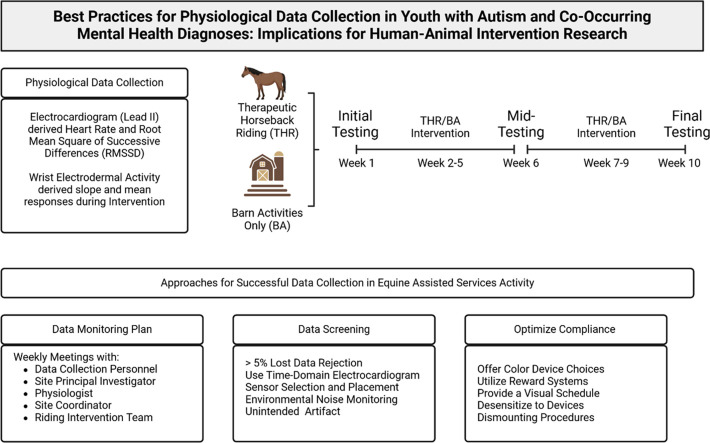

Specifications Table

**Table:** 

Subject area:	Psychology
More specific subject area:	Equine-Assisted Therapy
Name of your protocol:	Wearable Physiological Monitoring during Equine-Assisted Services
Reagents/tools:	Electrocardiogram Devices: The primary measure that needs to be captured are the R-R intervals for calculating heart rate variability measures (i.e., RMSSD). Multiple devices meet these needs and are research friendly which we have listed below. • BioPac BioNomadix Wireless ECG Monitor with a Physiology Data Logger (optional) • Shimmer3 ECG Unit • AdInstruments Equivital Wearable ECG • iWorx ROAM Wireless ECG • Zephyr Bioharness • Equivital Electrodermal Activity: Electrodermal sensors that allow the user to place gelled electrode patches in select locations should be utilized. Those that wrap the fingers should be avoided. • BioPac BioNomadix Wireless PPG and EDA Monitor with Physiologial Data Logger (optional) • Shimmer3 GSR/EDA Unit • AdInstruments Equivital Galvanic Skin Response Sensor • iWorx ROAM Wireless Biopotential & GSR Recorder Software: The analysis of dynamic ECG or EDA data should be performed on software that allows for appropriate cleaning of the data with ≤5 % artifact. • BioPac AcqKnowledge Software (ECG & EDA) • MATLAB Based Software (ECG & EDA) • LabView Based Software (ECG & EDA) • ADInstruments LabChart & HRV Analysis Software (ECG & EDA) • Kubios (ECG)
Experimental design:	This protocol outlines how to collect quality heart rate variability and electrodermal activity data during equine-assisted services. Special considerations for equipment placement, desensitization, and post-processing are required to reduce data loss associated with real-world dynamic physiological monitoring. The project this protocol was utilized in examined the impact of a 10-week therapeutic horseback riding program on the stress response in children with autism spectrum disorder. Physiological data was captured each week during the 1-hour intervention.
Trial registration:	NCT04606966
Ethics:	This project was approved by the principal investigator's institutional review board prior to conducting the study. In addition, parents provided consent for their child to participate in the study. This project involved the use of horses for the therapeutic intervention. A dedicated equine facility monitored the health and workload of all horses involved in the project in alignment with the guidelines developed by PATH International.
Value of the Protocol:	• Standardize electrodermal activity and electrocardiogram physiological analysis techniques for equine assisted services research. • Provide best practices for collecting physiological data in real-world equestrian environments with youth diagnosed with ASD.

## Introduction

In the U.S., there is a high prevalence of youth with autism spectrum disorder (ASD) [1] a diagnosis that is associated with unique impairments in social and communication abilities [2]. These impairments can negatively affect this population's ability to regulate their emotional responses when faced with the routine challenges of daily life [3,4] creating a greater need for effective intervention resources. Human-animal interaction (HAI) research has been shown to result in improvements in social, communication and behavior regulation needs of the ASD population [[5], [6], [7], [8], [9], [10]]. Equine-assisted services have emerged as a promising intervention for individuals with ASD, with a growing body of research exploring their efficacy [[11], [12], [13], [14], [15], [16], [17], [18]]. While both canine-assisted and equine-assisted services have been studied, there is an increasing focus on equine interventions for ASD. Recent research in this field has begun to incorporate physiological metrics to better understand the underlying mechanisms of observed positive outcomes. This shift towards physiological data collection is driven by the need to move beyond simple outcome measures and delve deeper into the biological processes at play during horse-human interactions. Increasing the quantity and quality of physiological data in equine-assisted intervention studies for youth with ASD can provide several advantages, including objective measurements, mechanism identification, and the potential for personalized interventions. Some of the physiological metrics being explored include heart rate variability, cortisol levels, electrodermal activity, and brain electrical activity (EEG) [19,20]. By incorporating these metrics, researchers aim to advance the field's understanding of how equine-assisted services impact individuals with ASD on a physiological level, potentially leading to more effective and targeted interventions.

Current HAI research highlights hypotheses about physiological benefits but notes a lack of empirical support due to methodological issues. While HAI is suitable for non-invasive, wearable physiological measurement, most studies rely on caregiver reports rather than physiological data [21,22]. Researchers employing physiological measures should consider population-specific challenges, such as device acceptance by ASD youth, and adequately train personnel in device application and data management. Developing a protocol that addresses these factors can enhance data quality and result accuracy in HAI studies. This paper provides the physiological data collection methodology from a large-scale randomized controlled trial with ASD youth ages 6 to 16 years aimed at identifying the physiological mechanisms resulting from engagement in a 10-week manual-based therapeutic horseback riding intervention compared to an active control (Clinical Trial # NCT04606966). The objective is to offer a systematic approach for future HAI researchers to collect viable cardiovascular and EDA data in youth with ASD in an equine intervention setting. The authors recommend future HAI researchers take a systematic approach to employing multiple physiological data collection methods (electrocardiogram (ECG) and electrodermal activity (EDA)) to account for measurement complications, maintaining high levels of data quality, and ensuring appropriate data monitoring with a population of youth with ASD.

To elevate HAI and improve scientific rigor, guidelines and lessons learned from other clinical research focus areas must be combined and critically examined in equine-assisted service environments. Although other clinical fields have developed methods to measure ECG and EDA data in dynamic movement environments, the inclusion of horses and youth with ASD pose unique challenges that must be explored to avoid misrepresentation of the physiological responses. That is, various forms of movement artifact, environmental factors, and physical exertion can significantly impact the data to reflect non-intervention related responses. The interpretation of non-intervention physiological related responses of the ECG and EDA signal as if they are what is occurring during equine-assisted services only serves as a disadvantage to the HAI field by spreading conflicting data. Therefore, this MethodX manuscript aims to serve as a catalyst for the HAI research to improve scientific rigor and accelerate the knowledge of field-based physiological responses during equine-assisted services (EAS) in youth with ASD.

## Methods

This paper provides the physiological data collection methodology of a large-scale randomized controlled trial (RCT) investigating physiological mechanisms in a 10 week manual-based (1R01HD097693–01A1; NCT: NCT04606966) [23] one-hour small group (2–4 participants) intervention (i.e., therapeutic horseback riding intervention compared to an active no-horse interaction Barn Activity (BA) control). Participants for this RCT included those with a study-confirmed diagnosis of ASD (ages 6 to 16 years) with co-occurring mental health symptoms. The challenge of collecting data while in a community therapeutic horseback riding center on horseback is the primary focus area of this methodological paper, particularly with study participants during the movement-based therapeutic horseback riding intervention.

### Initial screening

Participants’ ASD study diagnosis was confirmed using the ADOS-2 [24] and Social Communication Questionnaire-Lifetime version [25] and the presence of mental health symptoms was measured by the CASI-V [26]. Participants were excluded if they had prior horseback riding experience (i.e., received 10 consecutive weeks of therapeutic horseback riding within six-months prior to starting the study intervention). Participants taking medications or substances that could affect cardiovascular indices or EDA (e.g., amphetamines, anxiolytics, anticholinergics, beta blockers) were flagged for data analyses purposes (Table 1).

**Table 1:** Demographic data of study participants. includes *n* = 70 youth with autism spectrum disorder, who participated in the 10 week therapeutic horseback riding intervention. Nonverbal NVIQ: intelligence quotient; ADHD: Attention Deficit Hyperactivity Disorder; OCD: Obsessive Compulsive Disorder; PTSD: Post-Traumatic Stress Disorder.

THR participant Characteristics *n* = 70	N	Mean (SD)/Count ( %)	Range
Age	70	10.7 (2.9)	6.3–16.3
Sex	70	Male: 46 (66 %)	
		Female: 24 (34 %)	
Mean NVIQ	70	101.0 (22.3)	49.0–145.0
Prevalence of Psychological Comorbidity			
ADHD	70	53 (75.7 %)	
Anxiety	70	60 (85.7 %)	
Mood Disorder	70	35 (50 %)	
OCD	70	49 (70 %)	
PTSD	70	40 (57.1 %)	
Psychoactive Medications	69	47 (68.1 %)	
Baseline ABC—C			
Irritability	69	14.6 (8.1)	1–38
Lethargy	69	8.9 (7.0)	0–32
Stereotypy	69	3.4 (3.0)	0–12
Hyperactivity	69	17.5 (10.5)	0–46
Inappropriate Speech	69	3.2 (2.3)	0–10

### Riding center screening

A secondary screening was conducted at the riding center intervention site to orient the participant to center safety instructions and practice wearing the ECG and EDA equipment while engaging in a 10 min activity (e.g., riding on a horse with volunteer assistance). During this secondary screening, the optimal Motivation and Compliance strategies were determined (see Strategies for Improving Compliance and Data Quality below). Participants who could tolerate wearing the ECG and EDA devices as well as attending to adult direction were then scheduled for their randomized 10 week study intervention.

### Riding center site 10-week intervention groups

Each weekly session of the intervention, participants were told to arrive 20 min early to avoid rushing through the application of ECG and EDA equipment. For privacy, participants were given the option of placing their equipment on in a private room with their caregiver present. The ECG and EDA equipment was placed in the order of the child's preference to allow for greater compliance. Whenever possible, an established routine including the same environment and a consistent team member placing the ECG and EDA sensors was maintained across all 10 weeks of the intervention. After the equipment was placed, baseline data was collected while the participant was instructed to wait for the group to begin by sitting at an art and fidget toy materials table. During that same time, the caregiver moved to a separate room.

Physiological data (HR, HRV, and EDA) measurements were taken continuously during the entire intervention session. Study team members recorded the time of each activity for future data analysis including: (a) three to five minute seated art activity baseline before participants’ THR intervention; (b) 45 m45 min riding; (c) 15 m15 min grooming; and (d) 20 m20 mint intervention that included a five minute seated art activity. The Data Collection Personnel recorded the time and relevant activity levels for each of the baseline, intervention and post intervention activities as a reference to the device timestamps. In addition to timestamping the activities and device placement, the data collection personnel noted participants’ affect and any changes in volunteers, instructors, or horses the day of the intervention. Fidgeting with the devices and excessive movements by either the participant or the horse (i.e., running, jumping, trotting) were notated under the comments section of the physiological data log. Any unusual events that occurred leading up to or during the group were also noted under the comments section of the data log. The log category of “unusual events” was a place to record a variety of events that may affect physiological data such as a participant having a difficult school week, a car accident directly before group, or unusual events occurring in the arena during the intervention (e.g., horse having a startled reaction). If a participant fidgeted with a device, every effort was made by the volunteers, instructors, and study team members to redirect the participant's attention back to the intervention activity or to use alternative fidget material.

### Physiological data monitoring process

#### Physiological weekly data monitoring plan

The authors recommend that a physiological data collection monitoring plan be defined for HAI research protocols and that this plan includes weekly monitoring and troubleshooting of the data quality and collection methods. A data monitoring team should include the study data collection personnel, site principal investigator, and physiologist interpreting the physiological data. Weekly meetings should include a debrief of the data quality, participant device-wearing tolerance, device failures, and participant safety. Data monitoring meetings should also take place between intervention visits to allow data quality issues to be resolved before the upcoming intervention visit to reduce data loss. The overall goal of the physiological data monitoring plan is to ensure that there is an ongoing opportunity for the research team to successfully implement the data collection devices leading to optimal data quality.

#### Data monitoring team members

*Data Collection Personnel:* During participants’ intervention visits, study personnel person should notate timestamps for intervention activities, unique environmental factors (e.g., temperature and humidity), and participants’ behavioral characteristics (e.g., affect, atypical movements or interactions with intervention personnel) that occurred, which may impact data quality. After the participants’ intervention visit, the study personnel should send the de-identified raw data files and notations from each participant's visit to the Physiologist who should be blind to the participants’ intervention condition. In the weekly data monitoring meeting, this team member should provide feedback on the difficulties and successes regarding physiological device tolerance and the intervention session in a manner that does not lead to unblinding the physiologist.

*Site Principal Investigator:* During participant intervention visits, this study personnel should oversee and assure proper sensor placement, troubleshoot participant issues of device tolerance and ensure that the research protocol is followed according to the IRB-approved study protocol. During the weekly data monitoring meeting, the Site PI should oversee the Data Collection Researcher and Physiologist's discussion of the data and identify areas for improvement at the intervention site.

*Physiologist:* The data received by the Physiologist should be de-identified to ensure that the Physiologist remains blind to the intervention and participant characteristics. Therefore, the raw physiological data files the physiologist receives from the Data Collection personnel should be coded in a manner that does not indicate participant's intervention condition. The Physiologist should screen each participant's data for quality and unexpected issues in device function. This screening should occur before the weekly data monitoring meeting and before the coming weeks’ intervention visits.

*Site Coordinator/Riding Center Intervention Team:* The site coordinator should not attend the weekly meeting but receive feedback regarding suggested ways to improve data quality, device tolerance, and HAI-specific interventions. For example, if the sensors are getting caught and removed during the dismount phase, alternative approaches can be incorporated to reduce these occurrences. Feedback focus should be on the implementation of the modifications and approaches identified to increase data quality during the Data Monitoring Meeting. For example, if the data collection team identified a sensor disconnecting or movement artifact during the 45-minute intervention at week one, additional emphasis and techniques can then be used at week 2 to improve data quality.

#### Data quality screening

The Physiologist performing the weekly screenings should examine each data file for the percentage of data loss. If greater than 5 % of the data for the targeted analysis period has artifact or data loss, then the signal should not be used for further analysis. The Data Monitoring Team Members should determine potential causes of the data loss and develop strategies to mitigate data loss in subsequent intervention visits. A visualization of the data collected during the intervention should be created and presented by the Physiologist to the Data Monitoring Team to help identify the causes and implications of data artifact. Data loss is typically a result of either device failure, poor tolerance of the device, or a movement artifact that cannot be removed. Strategies for mitigating these types of data loss can be found in the Section Strategies for Improving Compliance and Data Quality. Only those files that have ≤5 % data loss should undergo second-order processing and final analysis.

#### Final data analysis

The final data analysis approach is dependent on the physiological data collected. In our HAI study (1R01HD097693–01A1; NCT04606966), we utilized EDA and ECG to monitor the participants’ stress responses during the intervention, in a community-based, real-world scenario. The analysis of signals collected during dynamic movement tasks, such as HAI research, require a great deal of care and additional rulesets for what data can be extracted from the signals and how external factors such as environment, compression, or movement may impact the signal. A trained human physiologist with experience performing physiological measurements during dynamic tasks is ideal for optimizing data quality and the validity of EDA and ECG responses during HAI research.

##### Electrocardiogram

A three-lead electrocardiogram should be collected using a Lead II configuration. The use of a three lead allows for clear identifications of the QRS complex even when movement artifact is causing shifts in the baseline isoelectric line of the signal. For example, rhythmic movement, mounting and dismounting, and leading all induce varying levels of movement artifact causing the ECG signal to have a wave like appearance. The primary components needed to capture R-R intervals and heart rate are still present but should undergo further signal processing to increase accuracy of the ECG-derived physiological metrics (Table 2).

**Table 2:** Representation of the percentage of usable data from *n* = 60 youth with ASD that completed the 10 week THR intervention. Baseline intervention data was collected in weeks one through Mid-point, intervention data was collected at weeks five through seven, and post-intervention data at weeks 8–10.

Availability of Data				
Physiology outcome	Any Data	Preintervention	Mid-point	post-intervention
ECG (*N* = 60)	45 (75 %)	45 (7 5 %)	39 (6 5 %)	45 (7 5 %)
EDA (*N* = 60)	48 (80 %)	48 (8 0 %)	43 (7 2 %)	48 (8 0 %)

The project's methodology was derived from utilized NI LabView Heart Rate Variability software with a Shimmer3 ECG unit, however, additional software and hardware options are provided in Specification Table. The hardware used to collect ECG in EAS research should allow for live data streaming and local data storage due to the dynamic nature of the research environment. For example, EAS research is typically performed in an open space including an arena, stables, and tack room where the human participant may be while wearing the ECG sensor. If a local data storage is not used and there is a loss in line of sight or too great a distance from the data collection station to the participant, there will be data loss. The software used for ECG analysis should have a detrend function that allows the Physiologist to reshape the data to simulate a consistent isoelectric line which increases the ability to identify the R-R interval. A detection threshold relative to the amplitude of each participants R-wave as the R-wave produces the greatest amplitude as the ventricles depolarize. The combination of these techniques allows the software to detect the peaks of each R-wave and record their timepoints for calculation of RMSSD and R-R intervals. After the detection of each R-wave, a visual inspection of the ECG signal should be performed and manually remove incorrectly identified R-waves typically due to movement artifact for shaking of the sensor which can result in large amplitude peaks. The heart rate and RMSSD can then be calculated. If >5 % of the ECG signal needed to be removed due to artifact, that signal should not be used for analysis.

Heart rate variability reviews are available which present flow charts for ECG acquisition, processing, and analysis as well as the physiological components captured from HRV which can be referenced as needed [27,28]. We found that measuring ECG during riding sessions in EAS research often results in large oscillations in the isoelectric line which impact the ability to accurately calculate many of the commonly utilized HRV components. In general, the time-domain measurements can be calculated after manipulating the signal as the detrend and artifact do not impact the time of the signal features. The most utilized HRV time-domain feature extracted is RMSSD, which reflects the beat-to-beat variance in the participants HR and is used to measure the vagally mediated changes occurring because of EAS. The HRV frequency-domain components should be used with caution as many of them require a steady isoelectric line to be accurately calculated and ensure the physiological adaptations are being captured and not simply artifact or signal manipulation.

##### Electrodermal activity

Electrodermal activity, also known as galvanic skin response, is a physiological signal that reflects the activity of the autonomic nervous system through the examination of skin conductance. Electrodermal activity has been used to examine arousal, affect, stress levels, and emotional regulation in children with ASD in a variety of environments and interventions [29]. An increase in acute stress response results in an increased EDA, while a reduction in stress or anxiety will be reflected in a decreased EDA. During EAS research, the participants often perform physical activity and are exposed to environmental conditions (i.e., heat, cold, humidity), both of which may impact the responses recorded using EDA. Whenever possible, intervention sessions should be performed in a climate-controlled environment and physical activity should be limited or standardized to reduce the impact these variations may have. In addition, the environmental temperature and humidity should be recorded during each intervention day.

The EDA signals are typically examined using tonic or fast varying phasic activity responses. During dynamic EAS research, a preference should be placed on utilizing tonic and not phasic responses. Specifically, caution should be used when examining the acute phasic activity of the EDA signal in EAS due to the higher potential for movement artifact and the need to know the exact timing of event (i.e., grooming or trotting). The tonic response, however, examines the changes to the signal overtime reducing the impact of artifact compared to a phasic activity response. The use of tonic responses during EAS can be performed over the duration of the intervention, such as the 45-min THR intervention, providing valuable physiological data that can be combined with the HRV data to examine the impact EAS has on the autonomic nervous system. The current study examined participants’ tonic responses during the structured THR intervention (45 min) as well as the time course of EDA responses by examining the first, middle, and last 15-minutes of the THR. Examining the slopes and mean conductance values for each timepoint across testing visits of the intervention can provide insights into the underlying psychophysiological responses associated with social and behavioral outcomes in youth with ASD.

#### Participant Descriptive Characteristics

##### Strategies for improving compliance and data quality

Collecting physiological data in non-laboratory, dynamic environments, such as EAS, is more difficult than in controlled laboratory settings. A primary concern of collecting physiological measures in real-world environments is the reliability of data. To improve reliability and quality of data, the processes outlined in Data Quality Screening and Final Data Analysis were utilized to ensure only high-quality data has been collected. In addition to these protocols, strategies for improving participant compliance and data quality can be administered at the research site through the implementation of motivation strategies, environmental considerations, human-horse interaction procedures, and physiological device acceptance and compliance.

##### Device acceptance strategies for ASD youth

Motivation strategies play a crucial role in improving engagement, compliance, and acceptance of the physiological monitoring devices in youth with ASD. The list below of suggested motivation strategies is in no order and all may be helpful for every participant. The research team conducting the data collection should be prepared and have available numerous motivational options to address the individualized needs to ensure quality data can be collected each week if a child has a dysregulation occur prior to or during the intervention that may impact the data. Additional strategies for successfully managing a population of youth with co-occurring mental health issues, as was the case in our current study, has been outlined by [30].

I. *Offer Choices:* Each child was given a choice of colored lanyard to attach to their EDA device as well as color-matched gloves to cover their EDA device. The approach has been shown to be effective with the majority of youth with ASD preferring colors in the blue-green spectrum and avoid yellow [31]. Often bright, intense colors result in overstimulation to youth with ASD and may reduce device acceptance [32]. Another choice that can be offered is the location where the EDA and ECG sensors are applied to the participant. For example, some participants preferred a private setting in an office while others preferred the classroom environment. Of note, older participants may feel more self-conscious of the ECG electrode placement over the chest and abdomen than the younger participants and therefore may prefer a private setting to don devices. Discussion with the parents or caregiver may help determine which environment may be optimal for each child.
II. *Implement a Reward System:* A reward system can be employed by the research team or parents of the child to increase their motivation to attend and engage in the research project. For example, a child may be offered their choice of a sticker or small fidget toys immediately following donning their devices. Additionally, a post-intervention reward activity can be offered that may be agreed upon between the caregiver and the participant.
III. *Provide a Visual Schedule:* It is important to have a visual schedule of the group activities for the day available to the participants to reference. This is typically an interactive schedule for the participant to note the progress through the class/session. The use of the visual schedules has been shown to reduce anxiety and improve compliance [33].

##### Device application and adherence strategies

Unused or extra equipment and consumables can be used to increase compliance by securing the device to the participant or desensitizing them to the electrodes or data acquisition units.

I. *Securing Devices:* Medical grade tape can be used to help secure electrodes and wires to the participant to limit movement artifact as well as unanticipated electrode removal. In addition, mesh arm wraps, or pre-wrap can be used to secure the data acquisition devices to the participant. This can help maintain device integrity during dynamic movements and secure the device during dismounting.
II. *Desensitizing:* The physiological device or a mock device should be worn by the participant during each visit, even if data collection is not being performed. This will standardize the visit and reduce variations in the participants’ experience which may cause dysregulation. In addition, some participants like to pick or play with the electrodes. To reduce this, additional, non-physiological, electrodes can be placed in an easily accessible spot to allow them to interact with the electrodes without impacting physiological electrodes.
III. *Maintaining Compliance:* One of the greatest challenges is continued compliance with the EDA and ECG devices throughout the session. Often participants will touch or pick at the electrodes, which impairs data quality. Volunteers overseeing the sessions can redirect the kid's hands and attention to another activity that is on task with the lesson (holding the reins, drawing/coloring pictures, holding miniature horses). Additional sensors that are not attached to the device can also be placed on the arm or chest, which they can pick without impacting data quality.
IV. *Dismounting Procedures:* Utilize an atypical therapeutic riding dismount when measuring physiological data. In a typical setting CTRI's are instructed to “slide” the participant down the right side of the horse for dismounting. This technique risks dislodging the physiological sensors placed on the front of the torso of the participant. An alternative technique would be to have the CTRI pull the participant away from the horse during dismount, to not compromise the lead placement while sliding down off the horse.

##### Environmental considerations

Environmental considerations are a necessary factor when collecting data in a community-based, non-laboratory setting. Considerations such as thermal and physical stress can be greater regulators to physiological markers than the study intervention. However, is not possible to address all environmental circumstances. As a result, any known limitation or impact that the environment may have on physiological metrics should be disclosed within the publication. This will allow other researchers to interpret physiological data within the context of the environment, often overlooked within EAS and real-world based research.

I. *Environmental Condition:* The temperature and humidity should be held relatively consistent and within normal limits for the location and acclimatization of the participants being studied. Exposure to extreme temperatures or large thermal shifts within a single session or across the 10 week intervention period can impact the interpretation of the results and should be avoided whenever possible.
II. *Riding Center Environment:* The riding center environment, such as the lighting, personnel, and setup should remain controlled and constant throughout every study visit to reduce novel experiences within the riding center from visit to visit.

#### Therapeutic riding center considerations

The therapeutic riding center is key to the successful implementation of the intervention and quality data collection in equine-assisted services environments. The therapeutic riding center should be included in the study team and provide feedback on proper equine welfare, participant engagement, and standardizing the intervention environment. A riding center should be screened for its ability to identify feedback loops of HAI between agitation of the horse and participants. Ideally, a facility who has prior experience with the population of interest (i.e., youth with ASD) is preferred as they will have adjusted to the unique needs associated with clinical populations. A successful and effective therapeutic riding center should have the following:

• PATH Intl Premier Accredited status or international accreditation
• Ten (+) years of experience in the EAS industry
• Infrastructure to support high quality research
• Highly trained certified therapeutic riding instructors
• Facility and physical spaces to hold consistent weekly programming
• Quality and quantity of equines
• Location and ease of accessibility to draw adequate numbers of participants
• Financial capacity to be a host site

## Funding

This study was supported by grant 1R01HD097693–01A1 from the National Institute of Health/National Institute of Child Health and Development (R.L.G.).

## CRediT authorship contribution statement

**Cory M. Smith:** Conceptualization, Methodology, Investigation, Data curation, Writing – original draft, Supervision. **Katharine Weimann:** Methodology, Investigation, Resources, Data curation, Writing – original draft. **Madison Widick:** Conceptualization, Methodology, Investigation, Resources, Data curation, Writing – original draft. **Tamara Merritt:** Methodology, Investigation, Resources, Writing – original draft. **Hannah Christensen:** Conceptualization, Investigation, Resources, Data curation, Writing – review & editing. **Matthew Siegel:** Conceptualization, Investigation, Writing – review & editing. **Zhaoxing Pan:** Formal analysis, Investigation, Writing – review & editing. **Robin L. Gabriels:** Conceptualization, Methodology, Investigation, Resources, Data curation, Writing – original draft, Supervision, Project administration, Funding acquisition.

## Declaration of competing interest

The authors declare that they have no known competing financial interests or personal relationships that could have appeared to influence the work reported in this paper.

## Data Availability

Data will be made available on request.
